# Does structured obstetric management play a role in the delivery mode and neonatal outcome of twin pregnancies?

**DOI:** 10.1007/s00404-023-07040-6

**Published:** 2023-04-28

**Authors:** Isabell Ge, Julia Meschede, Ingolf Juhasz-Boess, Mirjam Kunze, Filiz Markfeld-Erol

**Affiliations:** 1 Department of Obstetrics and Gynaecology, University Hospital Basel, Basel, Switzerland; 2 Breast Center, University Hospital Basel, University of Basel, Basel, Switzerland; 3 Department of Obstetrics and Gynecology, Medical Center, University of Freiburg, Freiburg, Germany; 4 Faculty of Medicine, University of Freiburg, Freiburg, Germany

**Keywords:** Twin pregnancy, Delivery mode, Predictive factors, Neonatal outcome, Obstetric management

## Abstract

**Purpose:**

While the optimal delivery method of twin pregnancies is debated, the rate of cesarean deliveries is increasing. This retrospective study evaluates delivery methods and neonatal outcome of twin pregnancies during two time periods and aims to identify predictive factors for the delivery outcome.

**Methods:**

553 twin pregnancies were identified in the institutional database of the University Women’s Hospital Freiburg, Germany. 230 and 323 deliveries occurred in period I (2009–2014) and period II (2015–2021), respectively. Cesarean births due to non-vertex position of the first fetus were excluded. In period II, the management of twin pregnancies was reviewed; adjusted and systematic training with standardized procedures was implemented.

**Results:**

Period II showed significantly lower rates of planned cesarean deliveries (44.0% vs. 63.5%, *p* < 0.0001) and higher rates of vaginal deliveries (68% vs. 52.4%, *p* = 0.02). Independent risk factors for primary cesarean delivery were period I, maternal age > 40 years, nulliparity, a history with a previous cesarean, gestational age < 37 completed weeks, monochorionicity and increasing birth weight difference (per 100 g or > 20%). Predictive factors for successful vaginal delivery were previous vaginal delivery gestational age between 34 and 36 weeks and vertex/vertex presentation of the fetuses. The neonatal outcomes of period I and II were not significantly different, but planned cesareans in general were associated with increased admission rates to the neonatal intensive care units. Inter-twin interval had no significant impact on neonatal outcome.

**Conclusion:**

Structured regular training of obstetrical procedures may significantly reduce high cesarean rates and increase the benefit–risk ratio of vaginal deliveries.

**Supplementary Information:**

The online version contains supplementary material available at 10.1007/s00404-023-07040-6.

## What does this study add to the clinical work

**Table:** 

This study shows that vaginal deliveries in twin pregnancies are safe and its success rate and safety can be enhanced through structured and regular updates of obstetrical concepts and procedures in obstetric departments. To lower the rate of cesarean deliveries in twin pregnancies, it is key to prevent the first cesarean birth.	

## Introduction

Worldwide, twin pregnancies account for 2–4% of all births [1]. Due to higher maternal age and a growing utilization of reproductive medicine, the number has risen since the last four decades [2, 3]. In 2021, Germany had an incidence of > 13,000 multiple pregnancies representing 1.7% of all births [4].

Risk-stratified analyses have shown variations of the mode of delivery within Europe in both singleton and twin pregnancies whereby in twins, the cesarean rates varied between 31.1% in Island and 98.8% in Malta. The Netherlands and France had significantly lower rates (43.9% and 54.8%) as compared to Germany and Italy with 74.8% or 85.6%, respectively [5]. According to a French prospective population-based study, vertex-first twins born between 32 and 37 gestational weeks by planned cesareans had higher composite neonatal mortality and morbidity rates with 5.3% versus 3.0%, respectively, as compared to vaginal deliveries [6]. These data suggest that national attitudes, guidelines, obstetric training skills and potentially financial incentives have a higher impact on the mode of delivery in twin gestations than any medical indication. Therefore, it was our hypothesis that the introduction of a strategy that involved senior obstetricians with a subspecialty in maternal–fetal medicine providing systematic training would increase the confidence that vaginal delivery of vertex-first twins can be easily performed and decrease the originally high elective cesarean rates.

In this retrospective study, we will assess the delivery methods and neonatal outcome of mono- and dichorionic twin pregnancies in a single institution. In this context, we will separately investigate two time periods with different clinical direction, beliefs, and expertise to explore whether a structured and systematic obstetric management may influence the rate of cesarean deliveries and neonatal outcome. Additionally, we intend to identify predictive factors for primary cesarean delivery and successful vaginal delivery and evaluate the neonatal outcome of each delivery mode as well as the obstetric management period.

## Materials and methods

### Study population and period

**Fig. 1:**
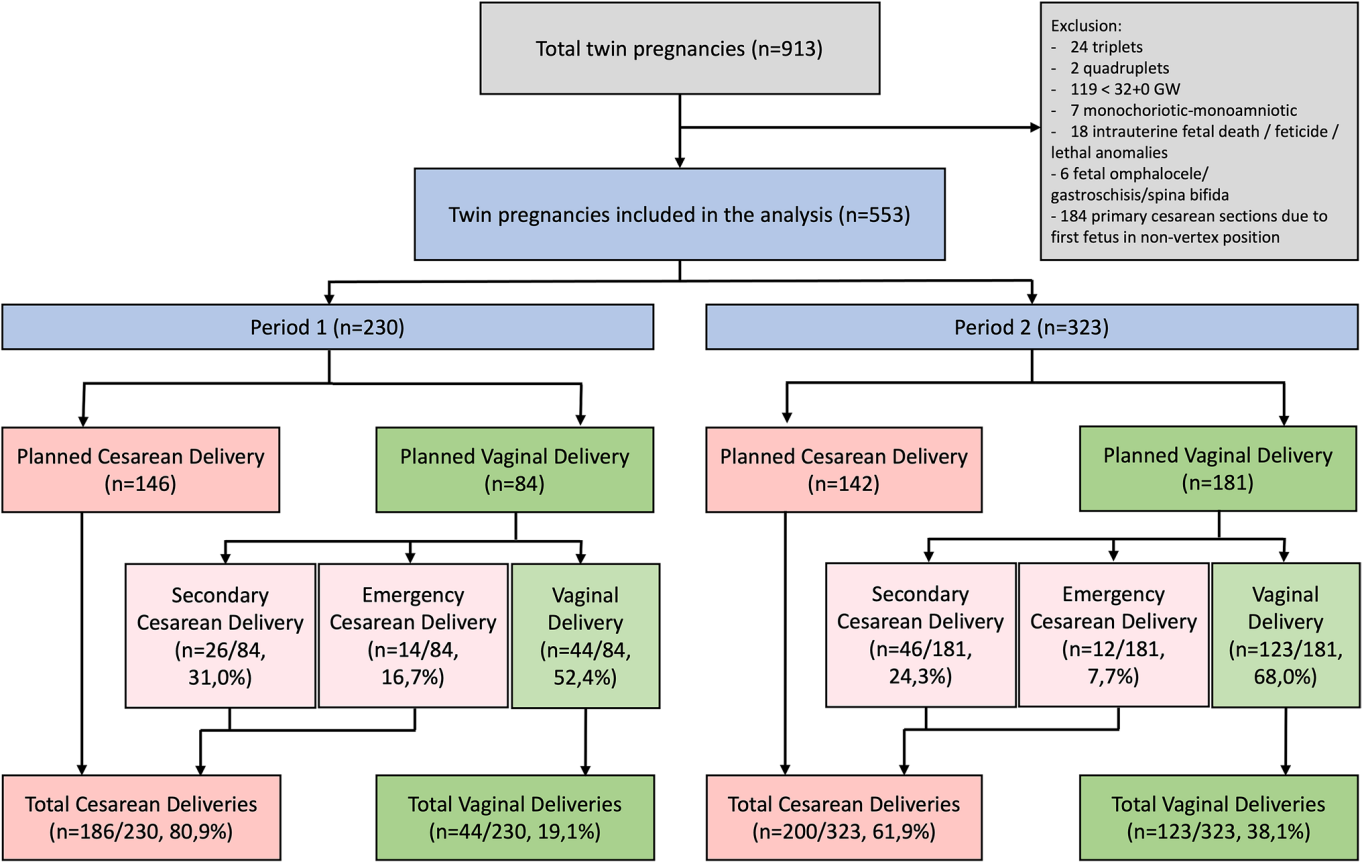
Study population of twin pregnancies categorized into two periods of obstetric management (period I = 2009–2014, period II = 2015–2021) and their respective distribution of cesarean and vaginal deliveries

We queried our institutional database on all multiple pregnancies starting at 32.0 weeks of gestation which were delivered between October 2009 and February 2021 at the University Women’s Hospital Freiburg. The following cases were excluded: triplets and quadruplets, monochorionic–monoamniotic twins, intrauterine fetal death, feticide, lethal congenital anomalies, omphalocele, gastroschisis, spina bifida and non-vertex position of the first twin. For details, see Fig. 1.

Two time periods were categorized: from October 2009 to December 2014 (period I) and from January 2015 to February 2021 (period II). Starting from period II, the management of twin pregnancies was reviewed and adjusted by two senior obstetricians with perinatal sub-specialization who implemented systematic training methods and standardized procedures. This change of policy was initiated by both after taking up leading roles in the department. They personally attended all twin deliveries at daytime and during their respective on-call duty. For the rest of the weekends and nighttime, they were available on standby to provide guidance for other senior physicians.

### Standardized delivery management of twin pregnancies

Compared to period I, vaginal twin deliveries were actively encouraged in period II. Vaginal deliveries were offered in uncomplicated twin pregnancies without contraindications for labor and when the first twin was presented in vertex position irrespective of the position of the second twin. Though not being an absolute indication for vaginal delivery, the estimated weight difference between both twins should not be significant, in contrast to period I preferring the weight discordance to not exceed 20% and the first twin being higher in weight. A vaginal delivery could be planned after one previous cesarean birth. If the first twin was in non-vertex presentation or if the patient had two or more previous cesarean births, a primary cesarean delivery was performed.

For a planned vaginal twin delivery, a team consisting of two obstetricians, one of which being a senior physician, a midwife and a midwife in training had to attend the birth. A neonatologist was available at all times. For potential (emergency) cesarean deliveries, operating staff including anesthesiologists and surgical nurses were on standby. To allow fast transfer, the operating room was situated in proximity right next to the delivery room.

For patients with planned vaginal delivery, the placement of an epidural anesthesia was recommended during the first stage of labor when no contraindications were present. This may facilitate the second phase of labor especially when the delivery of the second twin involves potential manipulation. After the delivery of the first twin, the uterus was manually stabilized and an abdominal ultrasound was immediately performed to verify the position and fetal heart rate of the second twin. If the fetus was in oblique or transverse position, an immediate artificial rupture of membranes and excessive iatrogenic procedures were refrained from which is in line with the suggestions by Arabin et al. [7]. Instead, the labor position was adapted to promote the engagement of the presenting part to either vertex or breech and its descent awaited. If the second fetus was in vertex or breech position and the labor proceeded physiologically, the descent of the head was also awaited without external force. It was aimed to achieve the delivery of the second twin within thirty minutes after the first twin. As long as the fetal heart monitoring was physiologic though, the wait could extend to up to one hour if necessary. Amniotomy was performed when the presenting part was in good contact with the pelvis and there was no risk of umbilical cord prolapse. Oxytocin was utilized restrictively and tocolysis was applied in case of pathological fetal heart rate changes. In case of pathological cardiotocography (CTG) or arrest of labor over an extended period, a vacuum or breech extraction, depending on the presentation and gestational age, may be applied.

### Statistical analysis

Statistical analysis was performed using SAS. We performed T-tests to compare normally distributed mean values as well as Mann–Whitney-*U*-tests for non-normally distributed values. The relationship between categorical variables was assessed using Fisher’s exact test and Pearson’s Chi-square test, respectively. Multivariate logistic regression analysis was used to identify independent variables predicting binary outcomes (such as primary cesarean delivery or successful vaginal delivery). Backward elimination with 20% significance level was used to adjust for potential confounders.

### Ethics statement

In accordance with the guidelines of the working group for the survey and utilization of secondary data (AGENS), no ethical approval is required for this study since it is a retrospective cohort study evaluating management and outcome of the department [8]. Still, the approval by our institutional ethics committee of the University Hospital Freiburg was received (21-1201).

## Results

### Descriptive analysis

A total of 913 cases of multiple pregnancies were identified, of which 553 were eligible for the analysis. 230 and 323 deliveries occurred in periods I and II, respectively (Fig. 1). Baseline characteristics of the study population (Table 1) showed no significant differences between the two time periods except for the delivery mode. Compared to period I, period II showed significantly lower rates of planned cesareans (44.0% vs. 63.5%, *p* < 0.001) and higher rates of vaginal deliveries (38.1% vs. 19.1, *p* < 0.001). The success rate of planned vaginal deliveries was also significantly higher in period II (68.0% vs. 52.4%, *p* = 0.02) with not only secondary cesareans (31.0% vs. 24.3%, *p* = 0.63) but also notably emergency cesareans significantly decreasing (7.7% vs. 16.7%, *p* = 0.01).

**Table 1:** Baseline characteristics of twin pregnancies during period 1 (2009–2014) and period 2 (2015–2021)

Variable	Period I Mean (range)/*n* (%)	Period II Mean (range)/*n* (%)	*p*-value
**Maternal age (years)**	33.1 (18–49)	32.4 (18–46)	0.14
**Gravidity**	1.9 (1–8)	2.0 (1–9)	0.18
**Parity**	1.5 (1–6)	1.6 (1–7)	0.09
**Maternal BMI (initial)**	24.1 (17–47)	23.9 (16–51)	0.76
**Maternal BMI (at birth)**	29.4 (21–55)	29.1 (19–62)	0.74
**Fetal weight (g)**			
1st twin	2442 g (1440–3575 g)	2431 g (660–3670 g)	0.74
2nd twin	2397 g (890–3610 g)	2358 g (870–3530 g)	0.46
**Fetal weight difference between 1st and 2nd twin (g)**			0.85
	308 g (0–1015 g)	307 g (0–1870 g)	
**Fetal sex**			
1st twin	Female 47.4%	Female 45.5%	0.73
	Male 52.6%	Male 54.5%	
2nd twin	Female 43.5%	Female 50.1%	0.14
	Male 56.5%	Male 49.9%	
**Planned delivery mode**			< 0.001
Planned cesarean delivery	146 (63.5%)	142 (44.0%)	
Planned vaginal delivery	84 (36.5%)	181 (56.0%)	
**Actual delivery mode**			< 0.001
Primary/secondary/emergency cesarean delivery	186 (80.9%)	200 (61.9%)	
Vaginal delivery	44 (19.1%)	123 (38.1%)	
**Vaginal delivery success**			0.02
Secondary/emergency cesarean delivery	40 (47.6%)	58 (32.0%)	
Sucessful vaginal delivery	44 (52.4%)	123 (68.0%)	
**Emergency cesarean at trial of vaginal labor**			0.01
Yes	14 (16.7%)	12 (6.6%)	
No	70 (83.3%)	169 (93.4%)	
**Maternal age (years)**			0.09
> 40	12 (5.2%)	7 (1.3%)	
≤ 40	218 (94.8%)	316 (97.8%)	
**Previous deliveries**			0.29
Primiparity	142 (61.7%)	173 (53.6%)	
Previous vaginal delivery	60 (26.1%)	101 (31.3%)	
Previous cesarean delivery	25 (10.9%)	43 (13.3%)	
Previous consecutive vaginal and cesarean delivery	3 (1.3%)	6 (1.9%)	
**Gestational age (weeks)**			0.20
32–34	27 (11.7%)	44 (13.6%)	
34–36	90 (39.1%)	145 (44.9%)	
≥ 37	113 (49.1%)	134 (41.5%)	
**Mode of conception**			0.75
Natural	150 (65.2%)	216 (66.9%)	
ART	80 (34.8%)	107 (33.1%)	
**Chorionicity**			0.17
Monochorionic	52 (22.6%)	91 (28.2%)	
Dichorionic	178 (77.4%)	232 (71.8%)	
**Presentation of the fetuses**			0.13
Vertex/vertex	161 (70.0%)	223 (69.0%)	
Vertex/breech	47 (20.4%)	82 (25.4%)	
Vertex/transverse	22 (9.6%)	17 (5.3%)	
Breech of 1st fetus	0 (0%)	1 (0.3%)	
**Fetal weight difference (percentage)**			0.69
< 20%	187 (81.7%)	268 (83.0%)	
≥ 20%	43 (18.3%)	55 (17.0%)	

*ART-assisted reproductive technologies

### Predictive factors

After adjusting for confounders, the obstetric management period was shown to be an independent predictor for planned cesarean delivery and successful vaginal delivery. Women with twin pregnancies in period I were over twice as likely to have a planned cesarean delivery (OR: 2.86 (95% CI 1.91–4.30), *p* < 0.0001)) and half as likely to have a successful vaginal delivery (OR: 0.5 (95% CI 0.28–0.89), *p* = 0.02) compared to women in period II (Table 2).

**Table 2:** Distribution of planned cesarean delivery and actual vaginal delivery according to obstetric management period (multivariate logistic regression)

	Yes, *n* (%)	No, *n* (%)	Odds ratio	95% confidence interval	*p*-value
Planned cesarean delivery*					
Period I	146 (63.5%)	84 (36.5%)	2.78	1.86–4.15	< 0.0001
Period II	142 (44.0%)	181 (56.0%)	1.00		
Successful vaginal delivery**					
Period I	40 (47.6%)	44 (52.4%)	0.49	0.28–0.89	0.02
Period II	58 (32.0%)	123 (68.0%)	1.00		

*Adjusted for age, parity, previous cesarean delivery, chorionicity, gestational age, fetal weight difference

**Adjusted for previous vaginal delivery, gestational age, presentation of the fetuses

Factors significantly associated with planned or primary cesarean delivery were maternal age above 40 years, nulliparity, a history with a previous cesarean, gestational age < 37 completed gestational weeks, monochorionicity and increasing birth weight difference per 100 g or > 20% (Table 3). Especially women who had a previous cesarean birth were 13 times more likely to undergo a planned cesarean delivery during the twin pregnancy. For birth weight difference between 1st and 2nd twin, the risk of a primary cesarean delivery increases by 16% with every 100 g and nearly threefold with ≥ 20% discrepancy. No significant associations were found with mode of conception, presentation of the fetuses and maternal BMI at birth.

**Table 3:** Distribution of planned delivery modes during both periods (*n* = 553, multivariate logistic regression): Predictive factors of a planned cesarean delivery

Variable	Planned cesarean delivery *n* = 288 (52.1%)	Planned vaginal delivery *n* = 265 (47.9%)	Odds ratio	95% confidence interval	*p*-value
**Maternal age (years)**					
≤ 40	271 (50.8%)	263 (49.3%)	0.13	0.03–0.62	0.01
> 40	17 (89.5%)	2 (10.5%)	1.00		
**Parity**					
Primiparity	183 (58.1%)	132 (41.9%)	3.12	1.97–4.95	< 0.0001
Multiparity	105 (44.1%)	133 (55.9%)	1.00		
**Previous deliveries**					
Previous cesarean birth	59 (76.6%)	18 (23.4%)	13.01	6.49–26.1	< 0.001
No previous cesarean birth	229 (48.1%)	247 (51.9%)	1.00		
**Gestational age (weeks)**					
32–33	55 (77.5%)	16 (22.5%)	5.65	2.79–11.4	< 0.0001
34–36	130 (55.3%)	105 (44.7%)	2.04	1.35–3.09	0.0008
≥ 37	103 (41.7%)	144 (58.3%)	1.00		
**Chorionicity**					
Monochorionic	86 (60.1%)	57 (39.9%)	1.94	1.21–3.13	0.006
Dichorionic	202 (49.3%)	208 (50.7%)	1.00		
**Presentation of the fetuses**					
Vertex/vertex	186 (48.6%)	197 (51.4%)	0.74	0.48–1.15	0.18
Other	102 (60.0%)	68 (40.0%)	1.00		
**Mode of conception**					
Natural	180 (49.2%)	186 (50.8%)	0.68	0.44–1.06	0.08
ART	108 (57.8%)	79 (42.3%)	1.00		
**Maternal BMI (at birth)**					
	Ø 28.9	Ø 29.5	0.99	0.95–1.02	0.45
**Fetal weight difference (continuous per 100 g)**					
	Ø 341 g	Ø 272 g	1.17	1.07–1.27	0.0005
**Fetal weight difference (categorical)**					
≥ 20%	72 (73.5%)	26 (26.5%)	2.95	1.68–5.16	0.0002
< 20%	216 (47.6%)	239 (52.5%)			

Factors significantly associated with successful vaginal delivery were previous vaginal delivery, gestational age between 34 and 36 weeks and vertex/vertex presentation of the fetuses (Table 4). Especially women with previous vaginal delivery were 7.9 times more likely to deliver twins vaginally. No significant impact on the rate of secondary cesarean delivery was shown with chorionicity, maternal age, parity, mode of conception, maternal BMI at birth and fetal weight difference.

**Table 4:** Distribution of planned vaginal delivery with or without vaginal delivery of both twins during both periods (*n* = 265, multivariate logistic regression). Predictive factors for a successful vaginal delivery

Variable	Planned vaginal delivery: secondary cesarean delivery *n* = 98 (37.0%)	Planned vaginal delivery: succesful vaginal delivery *n* = 167 (63.0%)	Odds ratio	95% confidence interval	*p*-value
**Maternal age (years)**					
≤ 40	96 (36.5%)	167 (63.5%)	> 999.9	< 0.001–> 999.9	0.98
> 40	2 (100%)	0 (0%)	1.00		
**Parity**					
Primiparity	69 (52.3%)	63 (47.7%)	1.29	0.41–4.07	0.67
Multiparity	29 (21.8%)	104 (78.2%)	1.00		
**Previous deliveries**					
Previous vaginal delivery	21 (17.8%)	97 (82.2%)	7.88	2.38–26.04	0.0007
No previous vaginal delivery	77 (52.4%)	70 (47.6%)			
**Gestational age (weeks)**					
32–33	6 (37.5%)	10 (62.5%)	1.14	0.34–3.82	0.83
34–36	31 (29.5%)	74 (70.5%)	1.84	1.00–3.39	0.049
≥ 37	61 (42.4%)	83 (57.6%)	1.00		
**Chorionicity**					
Monochorionic	20 (35.1%)	37 (64.9%)	0.88	0.44–1.76	0.72
Dichorionic	78 (37.5%)	130 (62.5%)	1.00		
**Presentation of the fetuses**					
Vertex/vertex	71 (36.0%)	126 (64.0%)	1.87	0.96–3.66	0.06
Other	27 (39.7%)	41 (60.3%)	1.00		
**Mode of conception**					
Natural	65 (35.0%)	121 (65.1%)	0.91	0.48–1.74	0.78
ART*	33 (41.8%)	46 (58.2%)	1.00		
**Maternal BMI (at birth)**					
	Ø 30.0	Ø 29.2	0.98	0.93–1.03	0.44
**Fetal weight difference (OR continuous per 100 g)**					
	Ø 266 g	Ø 274 g	1.06	0.92–1.22	0.44
**Fetal weight difference (categorical)**					
≥ 20%	8 (30.8%)	18 (69.2%)	1.79	0.66–4.85	0.25
< 20%	90 (37.7%)	149 (62.3%)			

*ART-assisted reproductive technologies

### Neonatal outcome

For neonatal outcome, we analyzed umbilical artery pH, APGAR score at 5 min and the transfer rate to the neonatal intensive care unit (NICU) in general and for pregnancies over 36 + 0th gestational weeks.

After primary cesarean delivery, the 2nd twin showed higher umbilical artery pH and APGAR score at 5 min compared to the other delivery modes; however, the transfer rate to the NICU was also higher for both twins (36.1%/40.3% for planned cesarean delivery vs. 15.3%/18.6% for vaginal delivery, see Table 5).

**Table 5:** Neonatal outcomes during both periods depending on delivery mode (*n* = 1106)

Twin	Planned cesarean delivery *n* = 576 (%) *n* = 310 for ≥ 36 + gestational weeks	Planned vaginal delivery, secondary cesarean delivery *n* = 170 (%) *n* = 131 for ≥ 36 + gestational weeks	Planned vaginal delivery, successful vaginal delivery *n* = 360 (%) *n* = 261 for ≥ 36 + gestational weeks	*p*-value
**Umbilical artery pH < 7.2**				
1st	4 (1.4%)	3 (4.2%)	6 (3.1%)	0.26
2nd	16 (5.6%)	15 (15.3%)	47 (28.3%)	< 0.0001
**APGAR score at 5 min < 7**				
1st	22 (7.6%)	7 (9.7%)	14 (7.3%)	0.79
2nd	20 (6.9%)	14 (14.3%)	25 (15.0%)	0.01
**Transfer to NICU**				
1st	104 (36.1%)	9 (12.5%)	39 (20.2%)	< 0.0001
2nd	116 (40.3%)	28 (28.6%)	31 (18.6%)	< 0.0001
**Transfer to NICU (≥ 36 + gestational weeks)**				
1st	6 (3.9%)	3 (5.2%)	7 (5.1%)	0.86
2nd	12 (7.7%)	9 (12.3%)	8 (6.5%)	0.34

For monochorionic twins, both primary and secondary cesarean deliveries showed higher NICU transfer rates for both twins compared to vaginal delivery. For dichorionic pregnancies, however, secondary cesarean deliveries showed the lowest transfer rate for the 1st twin. For the 2nd twin, vaginal delivery still resulted in the lowest rate of NICU transfer whereas primary cesarean delivery showed higher pH and APGAR scores. For pregnancies > 36 + 0th gestational weeks, there was no difference in the NICU transfer rate across all delivery modes (Table 6).

**Table 6:** Neonatal outcomes during both periods depending on chorionicity and delivery modes (*n* = 1106)

	Monochorionic				Dichorionic			
Twin	Planned cesarean delivery *n* = 172 (%)	Secondary cesarean delivery *n* = 36 (%)	Vaginal delivery *n* = 78 (%)	*p*-value	Planned cesarean delivery *n* = 404 (%)	Secondary cesarean delivery *n* = 134 (%)	Vaginal delivery *n* = 282 (%)	*p*-value
**Umbilical artery pH < 7.2**								
1st	2 (2.3%)	1 (6.3%)	0 (0.0%)	0.33	2 (1.0%)	2 (3.6%)	6 (4.0%)	0.17
2nd	5 (5.8%)	3 (15.0%)	6 (16.2%)	0.14	11 (5.5%)	12 (31.8%)	12 (15.4%)	< 0.0001
**APGAR score at 5 min < 7**								
1st	10 (11.6%)	3 (18.8%)	4 (9.8%)	0.64	12 (5.9%)	4 (7.1%)	10 (6.6%)	0.94
2nd	6 (7.0%)	2 (10.0%)	5 (13.5%)	0.51	14 (6.9%)	12 (15.4%)	20 (15.4%)	0.02
**Transfer to NICU**								
1st	42 (48.8%)	6 (37.5%)	7 (17.1%)	0.003	62 (30.7%)	3 (5.4%)	32 (21.1%)	0.0003
2nd	45 (52.3%)	9 (45.0%)	6 (16.2%)	0.0009	71 (35.2%)	19 (24.4%)	25 (19.2%)	0.005
**Transfer to NICU (≥ 36 + gestational weeks)**								
1st	2 (5.4%)	2 (22.2%)	2 (6.5%)	0.22	4 (3.4%)	1 (2.0%)	5 (4.7%)	0.69
2nd	3 (8.1%)	1 (10.0%)	4 (12.9%)	0.81	9 (7.6%)	8 (12.7%)	4 (4.4%)	0.16

In a subgroup analysis of successfully performed vaginal deliveries with the second twin being in non-vertex position, we evaluated the neonatal outcome based on the time interval between the delivery of the first and second twin (inter-twin delivery interval). Out of 167 successful vaginal births, 41 were delivered with the second twin being in non-vertex presentation. 25 occurred within an interval of < 30 min (61%) and 16 within an interval of > 30 min (39%). There were no significant differences in the neonatal outcome between the two groups (Table 7).

**Table 7:** Subgroup analysis of successful vaginal deliveries with second twin being in noncephalic position: Neonatal outcomes during both periods depending on inter-twin delivery interval (*n* = 82)

Twin	Interval < 30 min *n* = 50 (%)	Interval ≥ 30 min *n* = 32 (%)	*p*-value
**Umbilical artery pH < 7.2**			
1st	1 (4.0%)	2 (13.3%)	0.64
2nd	8 (32.0%)	7 (47.7%)	0.55
**APGAR score at 5 min < 7**			
1st	0 (0.0%)	2 (12.5%)	0.28
2nd	4 (16.0%)	5 (31.3%)	0.45
**Transfer to NICU**			
1st	5 (20.0%)	7 (43.75%)	0.20
2nd	5 (20.0%)	6 (37.5%)	0.38
**Transfer to NICU (≥ 36 + gestational weeks**)			
1st	1 (5.9%)	1 (11.1%)	1.00
2nd	2 (11.8%)	0 (0%)	0.77

Lastly, when comparing period I and period II, no significant differences in the neonatal outcome were observed (supplementary table).

## Discussion

### Principal findings

In our study, we demonstrated that the rate of cesarean deliveries of twin pregnancies decreased by 19% from 80.9% in period I to 61.9% in period II. By contrast, the success rate of planned vaginal deliveries significantly increased by 15.6% from 52.4% in period I to 68.0% in period II. After adjusting for other variables, we identified the obstetric management period as an independent predictor of planned cesarean delivery and successful vaginal delivery.

### Meaning of the findings

Considering that the average cesarean rate in Germany was shown to be around 75% [6], the cesarean rate in our department was initially above and later improved to below the German average without having an impact on short-term neonatal outcome. A significant decrease was achieved in planned cesareans but also in emergency cesareans, with the latter being an important obstetric outcome due to its association with increased maternal and neonatal morbidity and mortality [9, 10].

Other studies which evaluated interventions to reduce the rate of cesarean births included educational strategies for specialists and pregnant women and their families as well as managerial strategies such as pain-free labor or decision making for cesarean deliveries only by experienced physicians [11]. Another retrospective study reported a 33% relative reduction of cesarean deliveries after implementing a quality-improvement intervention comprised of modifications of the organization, staff training and unit policy [12].

While elective cesarean births in singletons are mostly requested due to psychological reasons or fertility issues [13], the data on elective cesarean deliveries on twin pregnancy are limited. Interestingly, in our study, the mode of conception, whether natural or via ART, did not have an influence on the mother’s choice (*p* = 0.18). Instead, the strongest predictors were previous cesarean delivery followed by gestational age from 32.0 to 33.6 weeks. Similar to our results, a retrospective study identified that women with a previous history of a cesarean birth and of older age (30 vs. 20 years) were more likely to undergo another cesarean delivery for their multiple pregnancy; however, the sample size of the study was very small (*n* = 47) [14].

For successful vaginal delivery, the strongest predictors were previous vaginal birth and vertex/vertex presentation of both twins, which have also been reported in the literature [15, 16]. In our study, mode of conception (natural vs. ART) and maternal age had no significant impact on the success of vaginal delivery. However, one study reported a higher vaginal birth rate with spontaneous conception [17], while another study identified that higher maternal age as well as maternal hypertensive disorder and diabetes decreased the likelihood of vaginal birth [16].

In our study, primary cesarean deliveries led to better neonatal pH and APGAR scores, but also higher NICU transfer rates in general. Our results are in line with several other studies showing a high incidence of respiratory morbidity and NICU admission of infants delivered by elective cesarean delivery [18].

### Clinical implications

According to the NICE (National Institute for Health and Care Excellence) guideline “Twin and triplet pregnancy” and the German guideline “Monitoring and care of twin pregnancies”, both the planned vaginal and cesarean deliveries are safe choices when certain conditions apply [19, 20].

Compared to vaginal labor, elective cesarean deliveries are associated with risks and complications such as postpartal hemorrhage [21], placental disorders [22], severe acute maternal morbidity [23], deep vein thrombosis, postpartal infection [9], longer in-patient stay [24] and impaired adaptation of the newborn [25]. Yet, increasing rates of cesarean delivery of twins had been reported. From 1990 to 2012, an overall increase of 23.5% was reported in Germany [26]. Similar trends were recorded in the United States [27].

Our results indicate that cesarean deliveries can be lowered when strategies or experienced attendance and supervision with regular teaching sessions and re-assurance of patients are systematically introduced—as in our center. Also, we identified having a history with a previous cesarean birth as a major risk factor for another cesarean delivery which implies, that preventing the first cesarean birth could be a key step to reduce the high rate of cesarean deliveries in twin pregnancies. Since fetal weight difference was identified as a risk factor for a planned cesarean, to further reduce cesarean rates, consideration should be given to the extent to which the estimated fetal weight difference between the two twins may influence the choice of delivery mode. According to the current guidelines, vaginal deliveries can be offered provided there is not a significant size difference between both twins. According to various sources, an estimated weight difference of 15–25% is considered as discordant [28, 29]. Our institution used to prefer a discordance of < 20% for vaginal deliveries in period I. However, based on the retrospective data available, twin discordance does not necessarily represent a contraindication for the trial of vaginal labor, even if the larger twin is the non-presenting twin. From the published data, weak evidence may support the consideration of cesarean delivery in extremes of discordance. From a practical standpoint, this may apply when the second twin is approximately > 40% larger than the presenting co-twin [30].

Concerning the ideal gestational age for vaginal delivery, our study identified that the success rate was at its highest during 34.0–36.6 weeks. A possible explanation could be the fetus’ increased resistance to labor stress during late preterm compared to lower gestational weeks and simultaneously being smaller in size and weight compared to higher gestational weeks, thus fitting through the birth canal more easily.

For neonatal outcome, elective cesarean sections showed the highest NICU transmission rate. Although birth asphyxia is less likely to occur when the fetus is not exposed to labor, the newborn may instead face higher difficulty of respiratory adaptation and the clearance of fluids in the lung [31]. This effect is most evident in early-term infants with surfactant deficiency. In our study, the NICU admission rate of infants born after 36.0 weeks was comparable between all three delivery modes. Interestingly though, for dichorionic pregnancies the NICU transfer rate for the first twin was remarkably lower after secondary cesarean delivery compared to the other delivery modes. A possible explanation could be that the first twin faced sufficient labor stress during the trial of labor, thus having less risk of respiratory adaptation difficulty compared to infants born by primary cesarean delivery. On the other hand, when the labor is terminated prematurely by secondary cesarean delivery, the risk of labor complications requiring postpartum neonatal care such as birth asphyxia or infection is also limited. For the second twin, the NICU admission rate after secondary cesarean delivery was considerably higher. This may be due to the fact that compared to the first twin, the second-born infant is faced with a significantly higher risk of respiratory distress syndrome which requires exogenous surfactant application [32, 33]. In a subgroup analysis, we additionally evaluated the inter-twin delivery interval since it is considered a risk factor for the short-term neonatal outcome of the second twin [34]. In our institution, the inter-twin delivery interval showed no significant impact. This is also reflected in our clinical practice as we limit iatrogenic measures after the delivery of the first twin and wait for the natural descent of the second twin regardless of its presentation, provided there is no fetomaternal harm and the fetal heart rate is physiologic. Similar to our study, other recent studies demonstrated that the short-term outcome of the second twin was not affected when the inter-twin delivery interval exceeded 30 min, raising the question of defining the optimal time frame for vaginal deliveries in twin pregnancies [35, 36].

### Research implications

Since this study is a retrospective analysis, further research should be dedicated to a prospective model in which a structured obstetric management for twin pregnancies is studied as an intervention. Also, maternal morbidity should be additionally assessed. Currently, there are intensive efforts within the German Workgroup Multiple Gestation to increase the skills and evaluate the results in the management of twin pregnancies (Hamza et al. unpublished).

### Strengths and limitations

Our study had several strengths including its large sample size and collection of data spanning over 10 years. Additionally, multivariate models were used to control for potential confounding. The weakness of the study lies in its retrospective model limiting the determination of a cause–effect relationship. Additionally, the study was limited to deliveries > 32 gestational weeks, a cutoff given by the NICE and German guidelines when vaginal birth can be offered [19, 20]. It should be noted though that there is evidence that vaginal deliveries can also be performed in vertex-first twins between 26 and 32 weeks with no negative impact on the outcome or significant differences in morbidity and mortality as compared to a primary cesarean [37, 38]. Lastly, for neonatal outcome, only immediate effects of the delivery were evaluated. Morbidity until discharge and long-term morbidity were not assessed.

## Conclusion

In our study, we have shown that obstetric management may influence the delivery mode of twin pregnancies. In our case, planned cesarean deliveries were reduced and the rate of successful vaginal labor was increased both significantly without impairment of the neonatal outcome. This concludes that vaginal deliveries in twin pregnancies are safe when no contraindications for labor apply. High rates of planned cesareans in general may be caused by multiple factors such as subjective indications suggested to the team and patients, lack of time and patience as opposed to a fast and scheduled delivery, financial incentives or the fear of litigation as seen in the practice of defensive medicine. Thus, this study marks the importance of structured and regular updates, training and review of concepts and procedures to maintain and improve the quality in an obstetrical department on a medical, educational and economical level.

### Supplementary Information

Below is the link to the electronic supplementary material.

Supplementary file 1 (DOCX 22 KB)

## Data Availability

The data presented in this study are availale on request from the corresponding author. The data are not publicly available due to privacy.
