# Supplementary material for: Does structured obstetric management play a role in the delivery mode and neonatal outcome of twin pregnancies?
Source: Arch Gynecol Obstet. 2023 Apr 28;309(4):1441–52. doi: 10.1007/s00404-023-07040-6 (PMC10894101; doi:10.1007/s00404-023-07040-6)
Supplement: Supplementary file 1 — (DOCX 22 KB) [file 404_2023_7040_MOESM1_ESM.docx]

*Supplementary table: Neoonatal outcomes depending on obstetric management period (n=1106)*

| Twin | Period I  n=460 (%) | Period II  n=646 (%) | p-value |
| --- | --- | --- | --- |
| Umbilical artery pH < 7,2 | | | |
| 1^st^ | 4 (1.7%) | 9 (2,8%) | 0.60 |
| 2^nd^ | 26 (11.3%) | 52 (16.2%) | 0.14 |
| APGAR score at 5 minutes <7 | | | |
| 1^st^ | 14 (6.1%) | 29 (9.0%) | 0.28 |
| 2^nd^ | 21 (9.1%) | 38 (11.8%) | 0.40 |
| Transfer to NICU | | | |
| 1^st^ | 64 (27.8%) | 88 (27.2%) | 0.96 |
| 2^nd^ | 71 (30.9%) | 104 (32.2%) | 0.81 |
| Transfer to NICU (≥ 36+ gestational weeks) | | | |
| 1^st^ | 7/146 (4.8%) | 9/205 (4.4%) | 1 |
| 2^nd^ | 12/146 (8.2%) | 17/205 (8.3%) | 1 |
